# Impact of Trainee Involvement on Outcomes in Acute Cholangitis: A Propensity-Matched Study in U.S. Hospitals

**DOI:** 10.7759/cureus.91741

**Published:** 2025-09-06

**Authors:** Karan Yagnik, FNU Payal, Sneh Sonaiya, Raj H Patel, Charmy Parikh, Anoohya Vangala, Malay Rathod, Pranav D Patel, Doantrang Du, Bradley D Confer, Ben Terrany, Dharmesh Kaswala, Harshit S Khara

**Affiliations:** 1 Internal Medicine, Monmouth Medical Center, Long Branch, USA; 2 Internal Medicine, University of Nevada Las Vegas School of Medicine, Las Vegas, USA; 3 Internal Medicine, St Mary Medical Center, Langhorne, USA; 4 Internal Medicine, Mercy Catholic Medical Center, Philadelphia, USA; 5 Gastroenterology, Hepatology and Nutrition, Geisinger Medical Center, Danville, USA; 6 Internal Medicine, RWJBarnabas Health, Long Branch, USA; 7 Gastroenterology and Hepatology, Monmouth Medical Center, Long Branch, USA

**Keywords:** acute cholangitis, nationwide inpatient sample (nis), teaching service, trainee physicians, trainee proficiency, us hospitals

## Abstract

Introduction: This study analyzed the outcomes of acute cholangitis (AC) in teaching hospitals, comparing July-September (new trainees) to April-June (experienced trainees). The July trainee transition may increase errors and inefficiencies despite diligent staff efforts. Outcomes were evaluated to determine the impact of trainee experience on patient care during these critical educational periods.

Method: This study analyzed adult hospitalizations (age >18) with AC in U.S. teaching hospitals using the National Inpatient Sample (2016-2020). A retrospective multivariate analysis was conducted using SAS 9.4 to evaluate inpatient mortality, length of stay (LOS), total hospitalization cost (THC, adjusted to 2020 USD), critical care interventions (e.g., intubation, ICU admission, CVC placement), endoscopic retrograde cholangiopancreatography (ERCP) utilization (with/without intervention), and ERCP timing from admission.

Results: A total of 12,360 patients were included in this analysis. Of those, 6355 (~51.42%) were admitted during July-Sept and 6005 (~48.58%) were admitted during April-June across a five-year period (2016-2020) in the United States. The median age of the patients was 73 years (IQR: 61-83) and 72 years (IQR: 61-83) for the period of July-Sept and April-June, respectively. Primary outcomes showed no difference in terms of in-patient mortality between the two groups (3.49% vs. 2.95%, p=0.1061). THC was slightly higher in the April-June group ($15,492 vs. $14,553) than in the July-Sept group. There were significantly higher rates of ICU admissions (6.26% vs. 4.83%, p=0.0009) and intubation (5.09% vs. 3.75%, p=0.0006) for patients who were managed during the months of July-Sept compared to those managed in April-June. However, there was no difference between two groups requiring central venous catheter (CVC) line placement (1.88% vs. 1.79%, p=0.7245) and ERCP utilization (85.17% vs. 85.25%, p=0.8941). Analysis revealed that the majority of patients had ERCP between 24 and 48 hours of admission in both groups (51.09% vs. 50.03%), than within 24 hours (25.42% vs. 25.02%) and after 48 hours (23.48% vs. 24.85%). However, there was no significant difference in timing between the two groups.

Conclusion: AC has no significant difference in mortality, LOS, THC, ERCP utilization, or ERCP timing between the two groups. However, patients managed from July to September experienced higher rates of ICU admissions and intubations compared to those managed from April to June. This may indicate a lower threshold for critical interventions due to trainee inexperience, with higher life-sustaining intervention rates driven by heightened patient safety measures to prevent errors.

## Introduction

Acute cholangitis (AC) is a medical emergency with a mortality rate of 4-10% with intervention and more than 50% without intervention [1,2]. Therefore, apart from conservative management with antibiotics and symptomatic care, timely biliary drainage with endoscopic retrograde cholangiopancreatography (ERCP) is necessary for source control either urgently (within 24 hours) or early (within 24-48 hours) [3,4].

In the United States, hospitals are classified as either teaching or non-teaching based on their role in training medical students, residents, fellows, and other healthcare professionals. Previous studies have produced mixed results regarding the impact of hospital teaching status on overall outcomes as well as disease-specific outcomes [5-9]. Our prior research indicated that teaching hospitals tend to have higher rates of mortality, septic shock, and ICU admissions compared to non-teaching hospitals [10]. We hypothesized that these differences could largely be attributed to the higher acuity of cases and varying levels of trainee proficiency.

In this study, we sought to investigate the influence of trainee experience on patient care by examining outcomes in AC at teaching hospitals. We compared patient outcomes between the first quarter of the academic year (July-September), when newly inducted trainees are integrated into the clinical setting, and the final quarter (April to June), when trainees typically possess greater clinical experience and autonomy in patient management.

## Materials and methods

Study design, database description, and data availability

This retrospective cross-sectional study analyzed hospitalization data from 2016 to 2020 using the Nationwide Inpatient Sample (NIS), which is part of the Healthcare Cost and Utilization Project (HCUP), developed and maintained by the Agency for Healthcare Research and Quality (AHRQ). The NIS is one of the largest publicly accessible all-payer inpatient databases, compiling data from hospital billing records submitted to statewide data agencies. It represents a stratified 20% sample of discharges from U.S. community hospitals, providing weighted estimates that reflect over 35 million hospitalizations annually, covering approximately 97% of the U.S. population.

The dataset includes comprehensive information on patient demographics, hospital attributes (such as geographic region and bed size), primary diagnoses, and procedures performed during admission. Since October 2015, all diagnoses and procedures in the NIS have been coded using the International Classification of Diseases, Tenth Revision, Clinical Modification/Procedure Coding System (ICD-10-CM/PCS). As the NIS is a de-identified, publicly available resource, its use does not require approval from an Institutional Review Board. Additional information on the NIS can be found at https://www.hcup-us.ahrq.gov.

Study population and variables

This study included all adults (aged >18 years) admitted to U.S. teaching hospitals with a primary diagnosis of acute cholangitis from 2016 to 2020, identified using ICD-10 codes from the NIS database. Patients under 18 years of age were excluded. The study population was further divided into two groups based on the time frame of admission: the first and last quarters of the academic year.

First Quarter

Patients admitted to U.S. teaching hospitals during the month of July, August, and September were included in this category. 

Last Quarter

Patients admitted to U.S. teaching hospitals during the month of April, May, and June were included in this category. Teaching hospitals were defined as hospitals with one or more Accreditation Council for Graduate Medical Education (ACGME)-approved residency programs, membership in the Council of Teaching Hospitals (COTH), or a ratio of full-time equivalent interns and residents to beds of 0.25 or higher.

Outcomes were categorized into two groups:

Primary outcomes: In-hospital mortality, hospital length of stay (LOS), and total hospitalization cost (THC), adjusted for inflation to 2020 values.

Secondary outcomes: Incidence of septic shock, ICU admission, requirement for intubation, central venous catheter (CVC) placement, ERCP utilization, timing of ERCP performed from admission.

All primary and secondary outcomes of AC were compared between the first and last quarters in U.S. teaching hospitals. Trends in all primary and secondary outcomes were analyzed over the period of 2016-2020. Moreover, hospitalization trend in relation to patient demographics, insurance status, median household income, hospital location (four main regions based on the United States Census Bureau), and presence of other comorbidities (congestive heart failure (CHF), hypertension (HTN), diabetes mellitus (DM), alcohol abuse) were analyzed.

Statistical analysis

All statistical analyses were performed using SAS version 9.4 (SAS Institute, Cary, NC). To ensure national representativeness, discharge-level weights provided by HCUP were applied in accordance with the sampling design of the Nationwide Inpatient Sample. Descriptive statistics were utilized to summarize patient and hospital characteristics. Continuous variables were expressed as means with standard deviations (SD), while categorical variables were presented as frequencies with corresponding percentages. Group comparisons for categorical and continuous variables were conducted using chi-square tests and independent t-tests, respectively.

To evaluate associations between demographic factors, comorbidities, hospital-level variables, and study outcomes, the Mantel-Haenszel chi-square test adjusted for the complex survey design was employed. Median values for age, length of stay (LOS), and total hospital charges were compared using weighted regression models. Temporal trends were assessed using chi-square tests for trend or t-tests, depending on the variable type.

To control for potential confounding, 1:1 propensity score matching was conducted based on baseline characteristics, including age, sex, insurance status, income quartiles, comorbid conditions, and hospital attributes. Multivariable logistic regression models were then used to estimate adjusted odds ratios (ORs) with 95% confidence intervals (CIs) for various outcomes. A two-tailed p-value ≤0.05 was considered statistically significant throughout the analysis.

Ethical compliance

Given that the NIS database is fully de-identified and lacks patient- and hospital-specific identifiers, this study was considered exempt from Institutional Review Board (IRB) oversight in accordance with institutional policies governing the use of HCUP-NIS data. The research was conducted in accordance with established ethical standards for human subjects research, including the principles outlined in the Declaration of Helsinki.

## Results

Patient characteristics and hospital characteristics

A total of 12,360 patients were admitted to U.S. teaching hospitals during our study period, of which 6355 (~51.42%) were admitted during the first quarter (July-Sept) and 6005 (~48.58%) were admitted during the last quarter (April-June) of the academic years. A five-year trend analysis showed that ~10% of patients were female for both quarters. Nearly half (>45%) of the population were falling in the age group of 65-84 years, with a mean age of 70 years (+/- 36.7 SD) for both quarters. The majority of the patients (~68%) were Caucasians and had Medicare (~66%) as their primary payer for both quarters of the academic years from 2016 to 2020. The majority of the population had median household income between the 26th and 75th percentile for both quarters of the academic years. Geographically, the majority of the hospitals were located in the south and northeast regions. The five-year trend of AC demographics is shown in Table 1 (first quarter) and Table 2 (last quarter).

**Table 1:** Trend analysis of demographics of AC during the first quarter of the academic year (2016-2020). AC: acute cholangitis.

Year	2016	2017	2018	2019	2020
Total	1065	1175	1340	1505	1270
Race					
White	655 (64.85%)	880 (79.28%)	915 (69.32%)	1020 (69.15%)	815 (65.99%)
Black	75 (7.43%)	40 (3.60%)	85 (6.44%)	95 (6.44%)	80 (6.48%)
Hispanic	150 (14.85%)	90 (8.11%)	180 (13.64%)	170 (11.53%)	205 (16.60%)
Asian or Pacific Islander	65 (6.44%)	70 (6.31%)	90 (6.82%)	105 (7.12%)	95 (7.69%)
Native American	15 (1.49%)	0 (0.00%)	5 (0.38%)	15 (1.02%)	25 (2.02%)
Other	50 (4.95%)	30 (2.70%)	45 (3.41%)	70 (4.75%)	15 (1.21%)
Primary payer					
Medicare	670 (63.21%)	815 (69.36%)	875 (65.30%)	995 (66.11%)	830 (65.35%)
Medicaid	150 (14.15%)	115 (9.79%)	170 (12.69%)	150 (9.97%)	120 (9.45%)
Private insurance	175 (16.51%)	185 (15.74%)	225 (16.79%)	235 (15.61%)	260 (20.47%)
Self-pay	65 (6.13%)	60 (5.11%)	70 (5.22%)	125 (8.31%)	60 (4.72%)
Region of the hospital					
Northeast	355 (33.33%)	360 (30.64%)	325 (24.25%)	480 (31.89%)	370 (29.13%)
Midwest	245 (23.00%)	225 (19.15%)	310 (23.13%)	350 (23.26%)	270 (21.26%)
South	300 (28.17%)	355 (30.21%)	335 (25.00%)	340 (22.59%)	295 (23.23%)
West	165 (15.49%)	235 (20.00%)	370 (27.61%)	335 (22.26%)	335 (26.38%)
Median household income national quartile for patient ZIP code					
0 to 25th percentile	255 (24.40%)	245 (21.03%)	255 (19.39%)	420 (28.47%)	280 (22.31%)
26th to 50th percentile (median)	265 (25.36%)	325 (27.90%)	360 (27.38%)	320 (21.69%)	315 (25.10%)
51st to 75th percentile	270 (25.84%)	325 (27.90%)	420 (31.94%)	380 (25.76%)	295 (23.51%)
76th to 100th percentile	255 (24.40%)	270 (23.18%)	270 (21.29%)	355 (24.07%)	365 (29.08%)
Female	545 (51.17%)	600 (51.06%)	670 (50.00%)	750 (49.83%)	615 (48.43%)
Age group					
18-44	110 (10.33%)	100 (8.51%)	145 (10.82%)	115 (7.64%)	110 (8.66%)
45-64	205 (19.25%)	285 (24.26%)	270 (20.15%)	320 (21.26%)	280 (22.05%)
65-84	575 (53.99%)	535 (45.53%)	615 (45.90%)	735 (45.84%)	625 (49.21%)
>84	175 (16.43%)	255 (21.70%)	310 (23.13%)	335 (22.26%)	255 (20.08%)

**Table 2:** Trend analysis of demographics of AC during the last quarter of the academic year (2016-2020). AC: acute cholangitis.

Year	2016	2017	2018	2019	2020
Total	1085	1140	1275	1295	1210
Race					
White	710 (69.95%)	760 (68.47%)	790 (63.97%)	880 (69.84%)	775 (66.24%)
Black	80 (7.88%)	60 (5.41%)	95 (7.69%)	105 (8.33%)	70 (5.98%)
Hispanic	134 (13.30%)	1650 (14.86%)	189 (15.38%)	120 (9.52%)	195 (16.67%)
Asian or Pacific Islander	55 (5.42%)	60 (5.41%)	105 (8.50%)	70 (5.56%)	99 (8.55%)
Native American	10 (0.99%)	10 (0.90%)	10 (0.81%)	15 (1.19%)	15 (1.28%)
Other	25 (2.46%)	55 (4.95%)	45 (3.64%)	70 (5.56%)	15 (1.28%)
Primary payer					
Medicare	705 (64.98%)	730 (64.04%)	775 (60.78%)	945 (72.97%)	785 (64.88%)
Medicaid	60 (5.53%)	105 (9.21%)	125 (9.80%)	75 (5.79%)	140 (11.57%)
Private insurance	259 (23.96%)	230 (20.18%)	310 (24.31%)	235 (18.15%)	195 (16.12%)
Self-pay	60 (5.53%)	75 (6.58%)	64 (5.10%)	40 (3.09%)	90 (7.44%)
Region of the hospital					
Northeast	274 (25.35%)	325 (28.51%)	345 (27.06%)	345 (26.64%)	325 (26.86%)
Midwest	245 (22.58%)	270 (23.68%)	269 (21.18%)	270 (20.85%)	255 (21.07%)
South	380 (35.02%)	330 (28.95%)	364 (28.63%)	375 (28.96%)	329 (27.27%)
West	184 (17.05%)	214 (18.86%)	295 (23.14%)	305 (23.55%)	300 (24.79%)
Median household income national quartile for patient ZIP code					
0 to 25th percentile	285 (26.51%)	245 (21.78%)	265 (21.03%)	310 (24.41%)	320 (26.67%)
26th to 50th percentile (median)	265 (24.65%)	305 (27.11%)	364 (28.97%)	299 (23.62%)	325 (27.08%)
51st to 75th percentile	229 (21.40%)	300 (26.67%)	334 (26.59%)	340 (26.77%)	260 (21.67%)
76th to 100th percentile	294 (27.44%)	274 (24.44%)	295 (23.41%)	320 (25.20%)	294 (24.58%)
Female	565 (52.07%)	625 (54.82%)	645 (50.59%)	645 (49.81%)	610 (50.41%)
Age group					
18-44	105 (9.68%)	129 (11.40%)	145 (11.37%)	85 (6.56%)	95 (7.85%)
45-64	274 (25.35%)	220 (19.30%)	289 (22.75%)	235 (18.15%)	304 (25.21%)
65-84	485 (44.70%)	515 (47.81%)	569 (44.71%)	645 (49.81%)	560 (47.51%)
>84	219 (20.28%)	244 (21.49%)	270 (21.18%)	330 (25.48%)	240 (19.83%)

A comparative analysis showed that more female patients were admitted during the last quarter of the academic year (52.55% vs. 49.15%, p=0.0003). There were also statistical differences between the two quarters for age groups, race, insurance status, median household income, and hospital region; however, clinical significance was small. Comorbidity comparison between the two quarters for the UGIB showed no significant difference for CHF, DM, alcohol abuse, and liver cirrhosis. More patients with HTN as a comorbidity were admitted to the hospital with AC during the first quarter of the academic year (69.71% vs. 65.42%, p<0.0001). This comparative analysis between the first and last quarters is shown in Table 3.

**Table 3:** Comparative analysis of demographics AC between the first and last quarters (2016-2020). AC: acute cholangitis; DF: degrees of freedom; CHF: congestive heart failure; DM: diabetes mellitus; HTN: hypertension.

Variables	First quarter (Jul-Sept)	First quarter (Jul-Sept)	Chi-square value (DF)	p-value
Female	2750 (49.15%)	2940 (52.55%)	12.9071 (1)	0.0003
Age group			16.4189 (3)	0.0009
18-44	535 (9.56%)	530 (9.47%)		
45-64	1125 (20.11%)	1215 (21.72%)		
65-84	2790 (49.87%)	2590 (46.29%)		
>84	1145 (20.46%)	1260 (22.52%)		
Race			11.8668 (2)	0.0026
White	3905 (69.79%)	3775 (67.47%)		
Black	325 (5.81%)	405 (7.24%)		
Other	1365 (24.40%)	1415 (25.29%)		
Primary payer			40.1322 (3)	<0.0001
Medicare	3720 (66.49%)	3660 (65.42%)		
Medicaid	615 (10.99%)	480 (8.58%)		
Private insurance	925 (16.53%)	1140 (20.38%)		
Self-pay	335 (5.99%)	315 (5.63%)		
Comorbidities				
CHF	980 (17.52%)	915 (16.35%)	2.6841 (1)	0.1040
HTN	3900 (69.71%)	3660 (65.42%)	23.4867 (1)	<0.0001
DM	1630(29.13%)	1720 (30.74%)	3.4510 (1)	0.0632
Alcohol abuse	155 (2.77%)	150 (2.68%)	0.0843 (1)	0.7716

Primary outcomes

Inpatient Hospital Mortality

**Figure 1:**
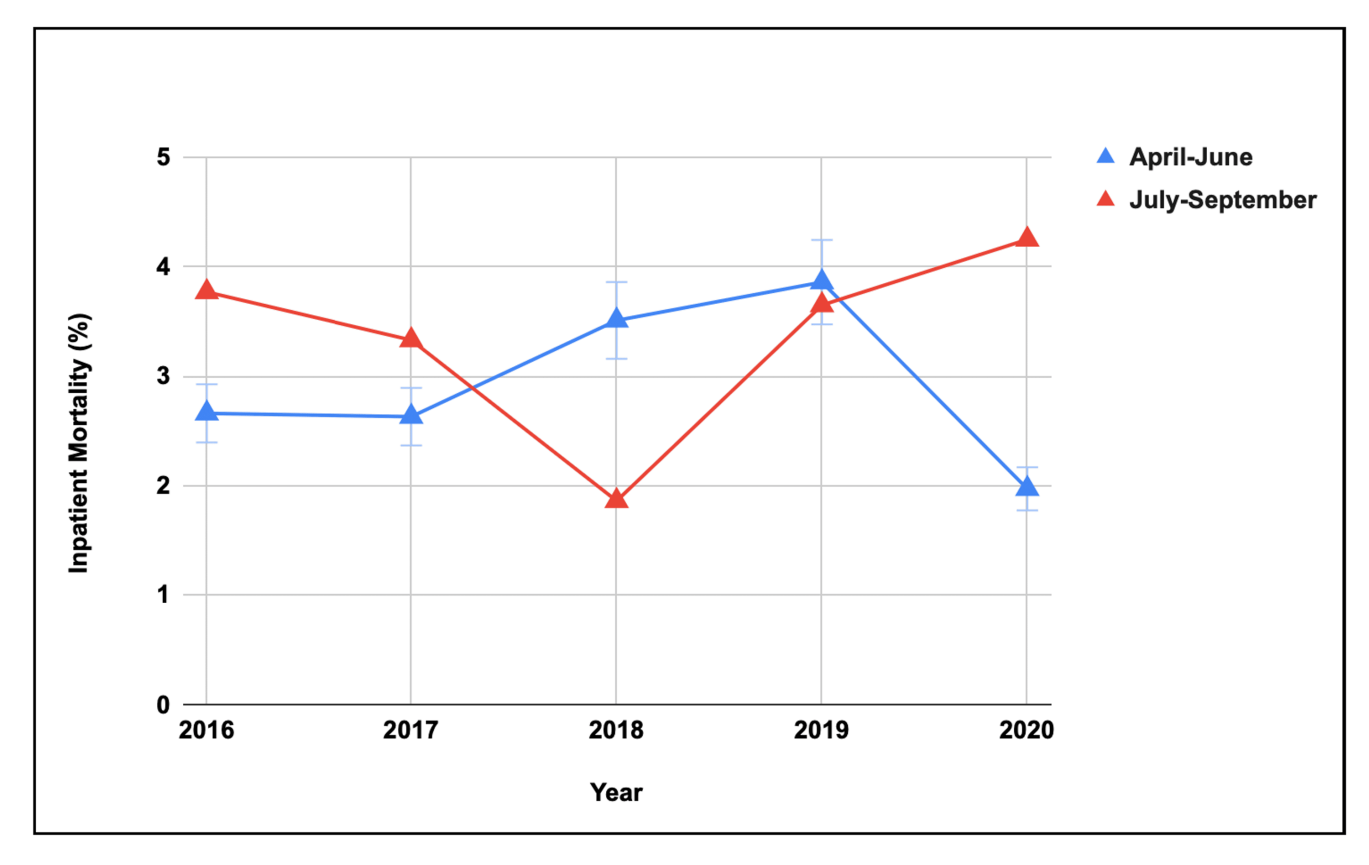
Comparison of inpatient mortality rate for AC in April-June versus July-September (2016-2020) in teaching hospitals in the United States. AC: acute cholangitis.

There was no significant difference in inpatient mortality for AC between the two quarters of academic years (Table 4). The five-year trend showed no difference in inpatient hospital mortality during the last quarter of the academic year, but it showed significant variability (p=0.0082) during the first quarter of the academic year (Figure 1).

**Table 4:** Comparative analysis of outcomes of AC between the first and last quarter (2016-2020). AC: acute cholangitis; DF: degrees of freedom; ICU: intensive care unit; CVC: central venous catheter; ERCP: endoscopic retrograde cholangiopancreatography.

Variables	First quarter (Jul-Sept)	First quarter (Jul-Sept)	Chi-square value (DF)	p-value
Mortality	195 (3.49%)	165 (2.95%)	2.6109 (1)	0.1061
Septic shock	855 (15.28%)	860 (15.37%)	0.0172 (1)	0.8956
ICU admissions	350 (6.26%)	270 (4.83%)	10.9280 (1)	0.0009
CVC placement	105 (1.88%)	100 (1.79%)	0.1242 (1)	0.7245
Intubation	285 (5.09%)	210 (3.75%)	11.8895 (1)	0.0006
ERCP	4765 (85.17%)	4770 (85.25%)	0.0177 (1)	0.8941
ERCP timings			0.5879 (2)	0.7453
<24 hours	302 (25.42%)	284 (25.02%)		
24-48 hours	607 (51.09%)	569 (50.13%)		
>48 hours	287 (23.48%)	282 (24.85%)		

Hospital LOS

The mean hospital LOS for AC was overall stable around six to seven days for both quarters of the academic year. The mean LOS was 6.38 days (±22.03) for the first quarter and 6.35 days (±12.57) for the last quarter of an academic year (Table 4).

Total Hospitalization Cost

The mean THC was $21,035 (±64,348) for the first quarter and $21,281 (±46,560) for the last quarter of the academic year (Table 4).

Secondary outcomes

A higher number of patients with AC had an ICU admission (6.26% vs. 4.83%, p=0.0009) and intubation (5.09% vs. 3.75%, p=0.0006) during the first quarter of the academic year compared to the last quarter during 2016-2020. However, there was no significant difference between the two groups for septic shock, CVC placement, and number of ERCP performed. Moreover, >50% of ERCP were performed between 24 and 48 hours for both quarters, and there was no difference in the timing of ERCP utilization between the groups (Table 4).

## Discussion

This study represents the first effort to examine the impact of trainee proficiency on patient care outcomes by comparing the management of AC in U.S. teaching hospitals during the first (July-September) and last (April-June) quarters of an academic year. Our analysis revealed no significant differences in inpatient mortality, the incidence of septic shock, or the placement of CVCs between the two quarters. However, patients in the first quarter exhibited a higher incidence of ICU admissions and intubation events.

Teaching hospitals, which serve as training grounds for residents, fellows, and medical students, are also tertiary referral centers, often tasked with managing underserved populations, high-risk cases, and patients with greater disease acuity [11-13]. While the acuity of cases should remain consistent throughout the year, external factors such as trainee experience may introduce variability. Our findings suggest that the significant increase in ICU admissions and intubation rates in the first quarter of the academic year may be attributed to the transition of new trainees.

The involvement of interns, residents, and fellows from various specialties could contribute to delays in familiarizing themselves with institutional protocols, slower order entry processes, and limited clinical experience, all of which can result in early or unnecessary interventions, such as ICU admissions and intubations, as observed in our study. These effects may be particularly pronounced during overnight shifts. Furthermore, restrictions on resident work hours and frequent handoffs between shifts can impact the continuity and quality of patient care [12,14].

Additionally, a systematic review has shown that the start of the academic year carries inherent risks, but as trainees progress through their training, outcomes generally improve [15]. Our study mirrored these findings, as the five-year trend did not reveal any difference in inpatient mortality during the last quarter of the academic year. However, significant variability in inpatient mortality was observed in the first quarter. Despite these differences, we found no variation in the timing or number of ERCP procedures performed between the two quarters, likely due to the fact that these decisions are primarily made by senior gastrointestinal attendings or consultants.

Strength and limitations

A key strength of this study is its novel contribution, offering the first comprehensive analysis of patient outcomes in AC in relation to trainee proficiency levels. Additionally, it presents national trends and clinical outcomes for AC from 2016 to 2020, utilizing data from the NIS. The NIS database is particularly advantageous due to its large sample size, which captures a broad representation of hospital discharges across the United States. Its standardized format, extensive range of variables, accessibility, and inclusion of cost-related data further enhance its utility for large-scale health services research.

This analysis employed multivariable logistic regression to estimate outcome odds, while 1:1 propensity score matching was used to account for baseline differences in patient demographics, comorbidities, and hospital-level characteristics. Although the findings provide valuable insights into evolving trends and healthcare outcomes associated with AC over a five-year period, certain limitations inherent to the study design remain.

The NIS database captures hospitalization-level data rather than tracking individual patients, which may introduce bias in estimating the prevalence of AC. This limitation could lead to underestimation by excluding cases managed in outpatient settings or overestimation by counting multiple hospitalizations for the same individual. Moreover, the absence of unique patient identifiers and granular clinical information restricts the assessment of prognostic variables that may influence mortality outcomes. In the context of ERCP, procedure timing was measured relative to hospital admission rather than emergency department (ED) presentation, which deviates from guideline-based benchmarks. Additionally, the database does not differentiate between the various therapeutic interventions performed during ERCP, as all procedures were grouped under a general ERCP code. Detailed information regarding lesion type and severity is also unavailable, limiting insights into disease-specific factors.

Another notable limitation pertains to the temporal analysis of trainee involvement. Ideally, comparisons would be made by tracking the same cohort of trainees from the first quarter of the academic year to its final quarter. However, the dataset spans from 2016 to 2020, thereby excluding first-quarter data for 2015 and last-quarter data for 2021. This temporal constraint prevents a longitudinal evaluation of individual trainee progression within a single academic year.

## Conclusions

This study provides valuable insights into the intersection of trainee proficiency and patient care in AC, highlighting subtle yet significant differences in clinical outcomes across the academic calendar. While overall mortality, ERCP utilization, and hospitalization costs remained consistent, the higher rates of ICU admissions and intubations during the first quarter suggest a potential "July effect," likely linked to the inexperience of newly inducted trainees. These findings underscore the delicate balance between medical education and patient safety in teaching hospitals. As healthcare systems evolve, the integration of robust supervision, structured handoffs, and standardized protocols will be essential to mitigating early trainee-related variability and ensuring optimal patient outcomes throughout the year. This study not only contributes to a deeper understanding of seasonal trends in the management of AC but also emphasizes the importance of continuous quality improvement in academic medicine to enhance both clinical training and patient care. Based on our study results, we recommend conducting further prospective blinded study to better understand the impact of trainees' proficiency on patient care across all medical specialties.
